# Association between SARS-CoV-2 and diabetes: A population-based cohort study using the UK CPRD GOLD database

**DOI:** 10.1007/s40200-026-02002-6

**Published:** 2026-07-08

**Authors:** P. Spivakovsky, N. L. Barclay, E. Burn, W. Y. Man, A. Delmestri, A. M. Jödicke, Daniel Prieto-Alhambra, M. Català

**Affiliations:** 1 Botnar Research Centre, Centre for Statistics in Medicine (CSM), NDORMS, University of Oxford, Old Rd, Headington, Oxford, OX3 7LD UK; 2 Dept. of Medical Informatics, Erasmus University Medical Centre, Rotterdam, Netherlands; 3 Dept. of Clinical Neurosciences, University of Oxford, Oxford, UK

**Keywords:** SARS-CoV-2, Diabetes, Association, Population, Cohort

## Abstract

**Purpose:**

Previous studies have reported contradictory findings about the possible association between SARS-CoV-2 and diabetes. We therefore conducted a cohort study to determine whether there is any association between SARS-CoV-2 infection and (i) new onset Type 1 diabetes and (ii) new onset Type 2 diabetes.

**Methods:**

We conducted a population-based cohort study on data from 01/01/2021 to 30/06/2022 using the UK Clinical Practice Research Datalink (CPRD) GOLD. In this database, the exposure cohort (Covid) was matched to the comparator cohort (No Covid), 1:1 with months as “enrolment” windows during 2021, without replacement. Large Scale Propensity Score matching was conducted using Lasso regression for variable selection. Then Poisson regression was used to examine the relationship between COVID-19 exposure and incident Type 1/Type 2 diabetes. A sensitivity analysis was performed by matching Covid cases with Negative tests.

**Results:**

161,870 COVID-19 patients were matched to 161,870 non-Covid controls; separately, 139,419 COVID-19 patients were matched to 139,419 test-negative participants. For Type 1 diabetes, the Poisson regression analysis gave an overall risk ratio of 1.27 (0.81–1.82); and a RR of 1.13 (0.66–1.84) when matching Covid with Negative tests. For Type 2 diabetes, we obtained an overall risk ratio of 1.10 (0.98–1.23); and a RR of 1.08 (0.96–1.20) when matching Covid with Negative tests.

**Conclusions:**

We do not find evidence of an association between SARS-CoV-2 infection and new onset of Type 1 or Type 2 diabetes. However, uncertainty remains due to the small number of events, especially in the case of Type 1 diabetes.

**Clinical Trial Number:**

Not applicable.

**Supplementary Information:**

The online version contains supplementary material available at 10.1007/s40200-026-02002-6.

## Introduction

 Type 1 (T1D) and Type 2 (T2D) diabetes are chronic metabolic disorders with a substantial impact on global health. The prevalence and incidence of both conditions have been steadily increasing over the past decades, particularly in children and young people, posing significant challenges to healthcare systems worldwide [1, 2]. Understanding the risk factors and complications of these diseases is crucial for effective prevention, management, and resource allocation.

The COVID-19 pandemic that swept the world in 2020 was particularly severe for patients with chronic conditions. Whilst there is evidence demonstrating that patients with diabetes are at greater risk of severe COVID-19 symptomology, severe and critical outcomes, COVID-19 hospitalisation and mortality [3–5], emerging evidence suggests that associations between diabetes and COVID-19 may be bidirectional [6, 7]. It is hypothesised that as the SARS-CoV-2 virus binds to angiotensin-converting enzyme 2 (ACE2) receptors in organs involved in metabolism, SARS-CoV-2 virus may have multiple adverse effects on glucose metabolism, potentially leading to new incident diabetes or complications of existing diabetes [6]. Indeed, a retrospective study of nearly 30 million patients in the USA demonstrated a 1.4-fold increased risk of developing new incident T1D in patients with COVID-19 compared to those without COVID-19 between the start of the pandemic and September 2021 [8]. Another study found even a 2-fold increase in the risk of developing diabetes following SARS-CoV-2 infection [9].

However, other publications have reported contradictory findings. A study of electronic health records in Scotland found no significant causal association between SARS-CoV-2 infection and development of diabetes in patients [10]. Another study including 428,650 patients with COVID-19 matched to controls found an increased incidence of diabetes shortly after the infection, but not in the long term (with some of the diagnoses of diabetes in the acute phase attributed to protopathic bias) [11].

Due to the heterogeneity of the results found in the literature, we conducted a cohort study with UK population data to examine this issue. Finding associations between SARS-CoV-2 and new onset T1D and T2D would help to increase our understanding of both diseases, and alert clinicians to monitor patients at high risk of disease. We therefore aimed to:

1. Evaluate the association between SARS-CoV-2 and new onset Type 1 diabetes.
2. Evaluate the association between SARS-CoV-2 and new onset Type 2 diabetes.

## Methods

### Study design and data source

We conducted a population-based matched cohort study. Participants were identified from a large representative source of pseudonymised UK primary care data: the Clinical Practice Research Datalink (CPRD) GOLD, release 2022/07 [12]. This database contains electronic health records of approximately 17 million patients, of which 3.1 million are currently active participants.

These data were mapped to the Observational Medical Outcomes Partnership (OMOP) Common Data Model, CDM version 5.3 [13, 14], to facilitate cohort curation and analyses.

### Study period and population

The study period covered dates when community testing for SARS-CoV-2 infection was routinely available and free in the UK: from 01/01/2021 to 30/06/2022.

The study population consisted of all people over 18 years of age registered within CPRD GOLD with at least 365 days of data availability and without diagnosis/medication of/for T1D/T2D any time before index date and without previous SARS-CoV-2 infection (identified by positive PCR test). The index date was defined as the date of COVID-19 diagnosis for the exposure cohort. The index date for people in the comparator cohorts was assigned at random within the same month following the same distribution as the index dates of the exposure cohort in “enrolment” windows during 2021.

### Study follow-up

Patients were followed up until the earliest of (1) outcome of interest (T1D/T2D), (2) the end of observation; (3) death; (4) transfer out of the database; (5) the end of data availability; (6) COVID-19 diagnosis in comparator cohort (leading to censoring of the matched pair).

### Selection of controls

The exposure cohort consisted of patients with a COVID-19 diagnosis recorded across the observation period, while the comparator cohort consisted of those patients without a COVID-19 diagnosis recorded anytime during this period.

To create the matched cohorts, the qualifying initial records were all people in the database with at least 1 year prior history, and no history of SARS-CoV-2 prior to January 1, 2021. Then, any patients with diabetes prior to January 1, 2021 were removed. Next, patients aged 18 and under were removed. Finally, the comparator cohort was matched to the exposure cohort, 1:1 with months as “enrolment” windows during 2021, without replacement. Large Scale Propensity Score (PS) matching was conducted using Lasso regression with five-fold cross validation for variable selection, and certain covariates (age, sex, obesity, and cohort start date) forced into the model. The PS distributions were examined after matching and standardised mean differences (SMD) calculated to assess covariate balance. All SMD values were found to be less than 0.1, indicating covariates were balanced after matching.

### Exposure definition

In this study, COVID-19 diagnosis was defined by positive PCR test for SARS-CoV-2. The PCR cycle threshold value for each test is not recorded in CPRD GOLD, but in the UK a typical PCR test for SARS-CoV-2 has a maximum of 40 thermal cycles [15]. Negative PCR tests for SARS-CoV-2 were also used in this study as a sensitivity analysis.

All codelists are available in the repository https://github.com/oxford-pharmacoepi/Covid-Diabetes-Association as JSON files.

### Outcome definition

In the UK, a patient is diagnosed with diabetes if [16]:

- An HbA1c test yields a result of 48 mmol/mol (6.5%) or above.
- A fasting plasma glucose test yields a result of 7.0 mmol/L or above.
- An oral glucose tolerance test yields a 2-hour glucose level of 11.1 mmol/L or above after consumption of a standard sugary drink.
- A random glucose test yields 11.1 mmol/L or above and the patient exhibits typical diabetes symptoms (such as increased urination, thirst, fatigue, weight loss or blurred vision).

Once a patient has been diagnosed with diabetes, it is classified as Type 1 or Type 2 by conducting a specialised test, typically a serum C-peptide test or a glutamic acid decarboxylase (GAD) test [16].

### Type 1 diabetes

In this study, new onset Type 1 diabetes following SARS-CoV-2 infection was defined as:

- A new diagnosis of T1D (incl. ketoacidosis) within 365 days following SARS-CoV-2 positive PCR test.
- No history of a diagnosis of T1D or Type 2 diabetes (T2D) any time before T1D diagnosis.
- No history of use of any antidiabetic drug any time before T1D diagnosis.
- No record of any non-insulin antidiabetic drug in the year after T1D diagnosis.

For the purposes of this definition, the look-back period was the patient’s entire medical history, from the moment the patient entered the database. The antidiabetic drugs considered in this study are provided as a codelist that is included in the Supplementary Materials, as well as in the aforementioned repository as a JSON file.

### Type 2 diabetes

New onset Type 2 diabetes following SARS-CoV-2 infection was defined as:

- A new diagnosis of T2D or first prescription of Metformin within 365 days following SARS-CoV-2 positive PCR test.
- No history of a diagnosis of T1D or T2D any time before T2D diagnosis.
- No history of use of any antidiabetic drug any time before T2D diagnosis.
- No previous diagnosis of polycystic ovary.

Again, the look-back period in this case was the patient’s entire medical history, from the moment the patient entered the database.

HbA1c measurements were only available for 39,156 patients in the COVID-19 group (24.2%) and 34,504 patients in the No Covid group (21.3%); but this data was taken into consideration when it was present in the patient’s record in order to verify the diagnosis of diabetes.

Phenotypes for new onset T1D and T2D were developed and tested before using the definition in this study. Plausibility tests were also conducted, including the review of patient characteristics (e.g. demographics, comorbidities and previous comedication) for individuals identified with new onset diabetes. The codelists are available in the repository https://github.com/oxford-pharmacoepi/Covid-Diabetes-Association as JSON files.

### Missing data

CPRD GOLD is a primary care database with high data quality and very low degree of missingness [17]. In particular, covariates such as age, sex, and cohort start date had 0 missingness in our study, as this information was available for all participants. For other covariates, such as positive/negative PCR tests for SARS-CoV-2, and outcomes, Type 1 and Type 2 diabetes, missingness was estimated to be very low, as all tests and diagnoses in the NHS primary care must be recorded by the GP in the patient’s electronic health record, so the probability that a test or diagnosis occurred but was not recorded due to human error is very small [17].

Nevertheless, our study was conducted with the assumption that a small proportion of data could still be missing at random (MAR), and as a result the methods were chosen to be robust to this level of missingness. In particular, five-fold cross validation was implemented for variable selection in the Lasso regression, and a sensitivity analysis was conducted to check the validity of the results obtained.

However, due to the high quality of the dataset, missingness itself was not adjusted for explicitly in the matching or propensity score model, as the small proportion of missing data was considered unlikely to have a significant impact on the results of the study.

Of course, a key limitation of this study, and of any study using real-world data, is that we only work with the data that is available in the database; thus, until a disease has been diagnosed and entered into the database, we are forced to treat the absence of a diagnosis as absence of the disease. Thus, the No Covid control group for instance may have included some patients with undiagnosed COVID-19 that had not yet been tested; however, this was likely a very small proportion of the participants, as SARS-CoV-2 testing was freely available in the UK during the study period.

### Statistical analysis

We compared rates of incident T1D and T2D between the exposure and comparator cohorts. Statistical analyses were performed using R version 4.2.3 [18] and consisted of the following:

#### Summary descriptive statistics

Both cohorts were characterised on a number of baseline characteristics, including age, sex, comorbidities, comedications, obesity. Characterisation was done using the standardised pipeline described in the CohortCharacteristics package [19].

#### Poisson Regression

Poisson regression was used to examine the relationship between COVID-19 exposure and incident T1D or T2D across multiple time windows after index date: 0–30 days, 31–90 days, 91–180 days, 181–365 days.

#### Cox-proportional hazard regression

Hazard ratios for each outcome (T1D and T2D) were calculated using Cox proportional hazard models clustered by matched pairs, and the Kaplan-Meier survival curves plotted.

#### Sensitivity analysis

The same analysis was carried out matching Covid with Negative PCR test and compared to the results obtained matching Covid with No Covid.

All code used in this study is available in the repository https://github.com/oxford-pharmacoepi/Covid-Diabetes-Association, and full results including tables/visualizations are available in the interactive web application at https://dpa-pde-oxford.shinyapps.io/Covid-Diabetes.

## Results

The characterisation of the matched cohorts is shown in Table 1 (for the characterisation prior to matching, see Table 3 in the Appendix). As observed, the COVID-19 and control matched cohorts were comparable in terms of demographics and comorbidities. The median follow-up time was 236 days for the COVID-19 group, and 237 days for the control group. Standardised mean differences were not included in Table 1 due to space limitations, but are readily available in the interactive web application mentioned above. All SMD values were found to be less than 0.1 for the matched cohorts.

**Table 1:** Characterisation of matched cohorts in the CPRD GOLD database

	No Covid or Negative Test		Covid	
	Covid	No Covid	Covid	Negative Test
Number of Subjects	161,870	161,870	139,419	139,419
Number of Records	161,870	161,870	139,419	139,419
Sex: Female	87,899 (54%)	87,899 (54%)	74,179 (53%)	74,179 (53%)
Male	73,971 (46%)	73,971 (46%)	65,240 (47%)	65,240 (47%)
Age: Median (q25-q75)	42 (31–54)	42 (31–54)	43 (31–56)	43 (31–56)
0 to 19	3,251 (2%)	3,251 (2%)	2,575 (2%)	2,575 (2%)
20 to 39	68,260 (42%)	68,260 (42%)	55,806 (40%)	55,806 (40%)
40 to 59	65,193 (40%)	65,193 (40%)	54,740 (39%)	54,740 (39%)
60 to 79	22,323 (14%)	22,323 (14%)	23,959 (17%)	23,959 (17%)
80 to 150	2,843 (2%)	2,843 (2%)	2,339 (2%)	2,339 (2%)
Comorbidities: Diabetes	0 (0%)	0 (0%)	0 (0%)	0 (0%)
HIV	76 (0%)	69 (0%)	78 (0%)	73 (0%)
Stroke	952 (1%)	1,014 (1%)	837 (1%)	845 (1%)
Myocardial infarction	1,352 (1%)	1,269(1%)	1,158 (1%)	1,162 (1%)
Inflammatory bowel disease	1,191 (1%)	1,080 (1%)	968 (1%)	1,109(1%)
Osteoporosis	1,405 (1%)	1,471 (1%)	1,341 (1%)	1,500 (1%)
Rheumatoid arthritis	855 (1%)	748 (1%)	756 (1%)	780 (1%)
Chronic liver disease	271 (0%)	361 (0%)	243 (0%)	245 (0%)
Chronic kidney	2,761 (2%)	2,635 (2%)	2,476 (2%)	2,426 (2%)
Heart failure	752 (0%)	732 (0%)	641 (1%)	644 (1%)
Venous thromboembolism	2,627 (2%)	2,532 (2%)	2,270 (2%)	2,326 (2%)
Hypothyroidism	4,625 (3%)	4,751 (3%)	3,696 (3%)	3,926 (3%)
Depressive disease	24,108 (15%)	24,656 (15%)	19,132 (15%)	20,023 (15%)
Anxiety	30,220 (19%)	30,948(19%)	23,747 (19%)	24,798 (19%)

**Fig. 1:**
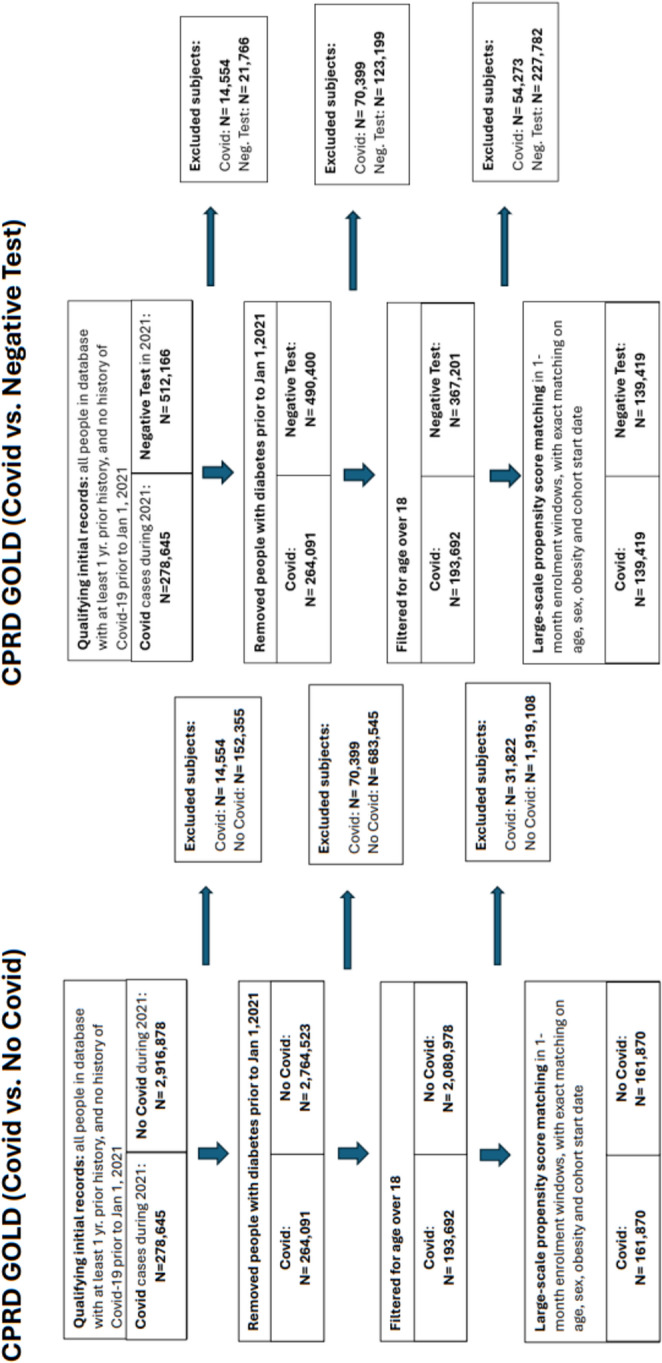
Schematic diagram showing the study design and attrition

The study design and attrition are shown schematically in Fig. 1. When matching Covid with No Covid, the matched cohorts included 161,870 patients with Covid, and 161,870 with No Covid; when matching Covid with Negative test, there were 139,419 patients in each cohort.

The counts of COVID-19 cases, Type 1 and Type 2 diabetes after matching are shown in Table 2 (for the counts prior to matching, see Table 4 in the Appendix).

**Table 2:** Patient counts for COVID-19 cases, Type 1 and Type 2 diabetes in the matched cohorts

	No Covid or Negative Test			Covid		
	*N*	Type 1 Diabetes	Type 2 Diabetes	*N*	Type 1 Diabetes	Type 2 Diabetes
CPRD GOLD (Covid vs. No Covid)	161,870	21	443	161,870	23	452
CPRD GOLD (Covid vs. Negative Test)	139,419	19	402	139,419	20	395

**Fig. 2:**
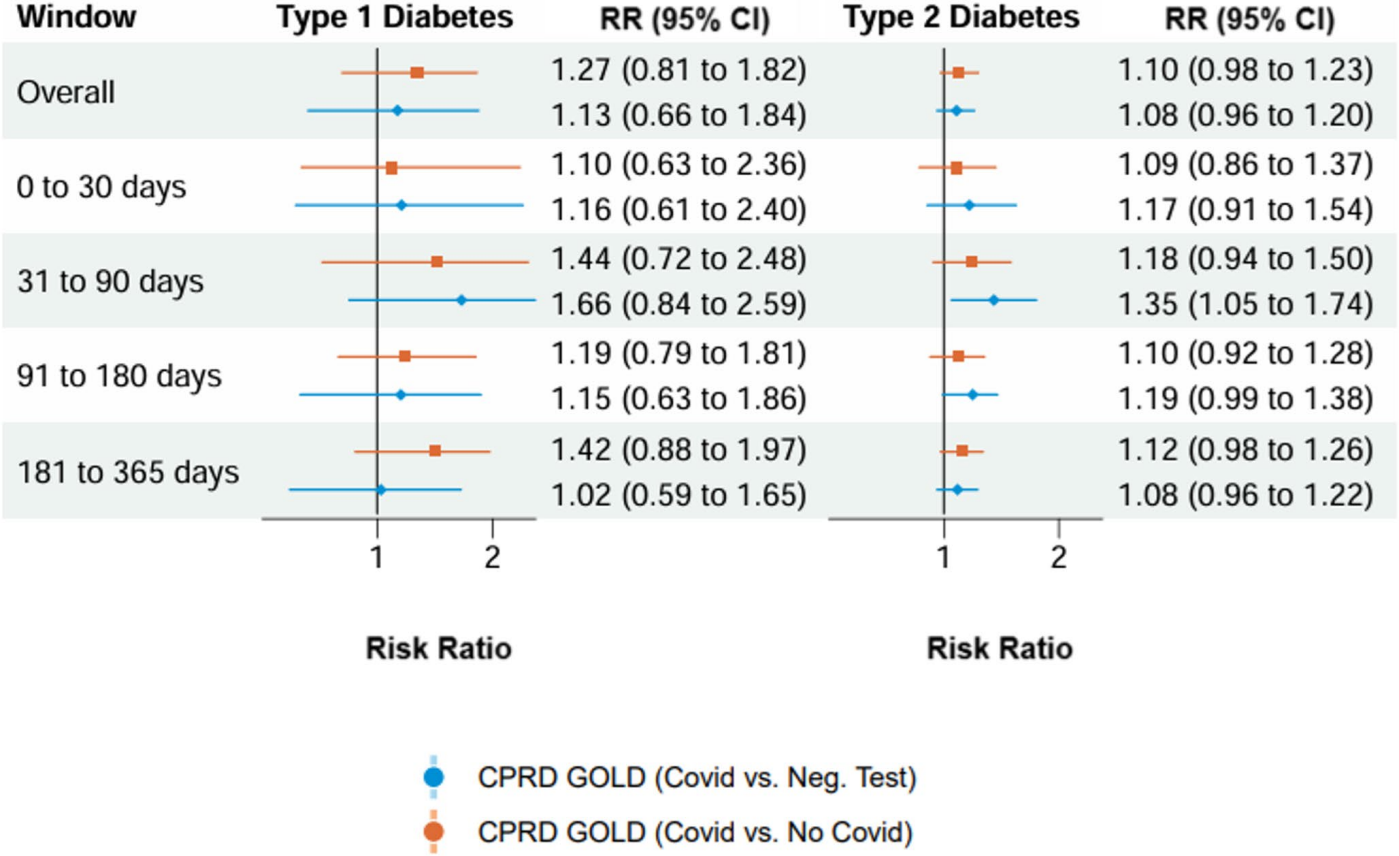
Results of Poisson regression for different time windows

The results of the Poisson regression for different time windows are shown in Fig. 2. In particular, for Type 1 diabetes, when matching Covid with No Covid, we obtained an overall risk ratio of 1.27 (Lower 95% CI, 0.81- Upper 95% CI, 1.82); a RR of 1.10 (0.63–2.36) for the 0–30 days time window; a RR of 1.44 (0.72–2.48) for the 31–90 days time window; RR of 1.19 (0.79–1.81) for the 91–180 days time window; and finally 1.42 (0.88–1.97) for the 181–365 days time window.

When considering the matched cohort of Covid with Negative test, we obtained an overall risk ratio of 1.13 (0.66–1.84); a RR of 1.16 (0.61–2.40) for the 0–30 days time window; a RR of 1.66 (0.84–2.59) for the 31–90 days time window; RR of 1.15 (0.63–1.86) for the 91–180 days time window; and finally 1.02 (0.59–1.65) for the 181–365 days time window.

For Type 2 diabetes, when matching Covid with No Covid, we obtained an overall risk ratio of 1.10 (Lower 95% CI, 0.98- Upper 95% CI, 1.23); a RR of 1.09 (0.86–1.37) for the 0–30 days time window; a RR of 1.18 (0.94–1.50) for the 31–90 days time window; RR of 1.10 (0.92–1.28) for the 91–180 days time window; and finally 1.12 (0.98–1.26) for the 181–365 days time window.

**Fig. 3:**
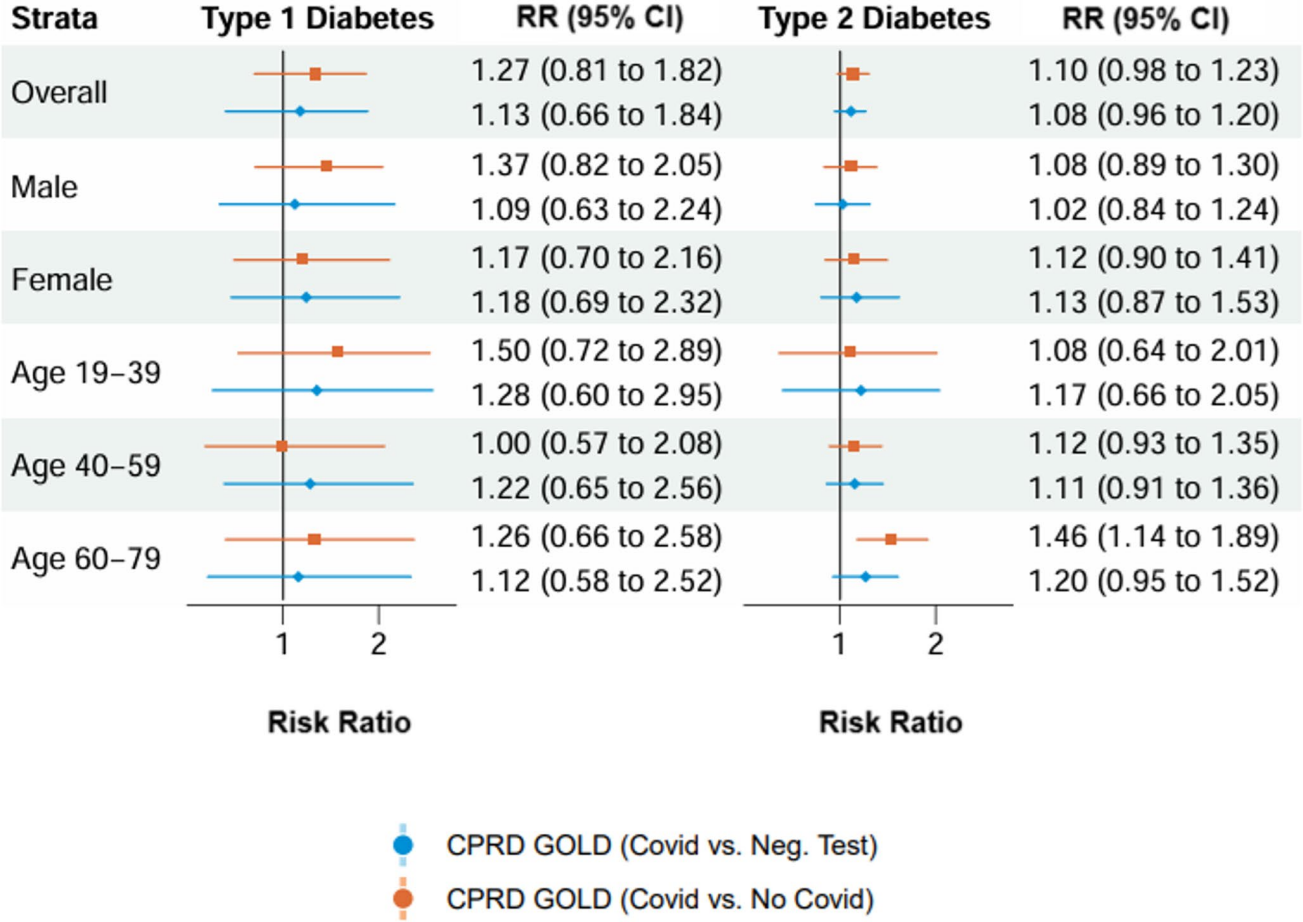
Results of poisson regression stratified by age and sex

When considering the matched cohort of Covid with Negative test, we obtained an overall risk ratio of 1.08 (0.96–1.20); an RR of 1.17 (0.91–1.54) for the 0–30 days time window; RR of 1.35 (1.05–1.74) for the 31–90 days time window; RR of 1.19 (0.99–1.38) for the 91–180 days time window; and finally, 1.08 (0.96–1.22) for the 181–365 days time window. The results of the Poisson regression analysis stratified by age and sex are shown in Fig. 3.

**Fig. 4:**
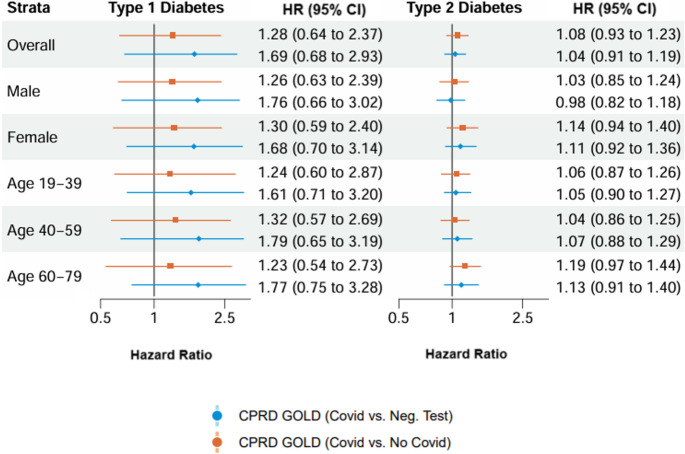
Hazard ratios by strata for Type 1 and Type 2 diabetes

**Fig. 5:**
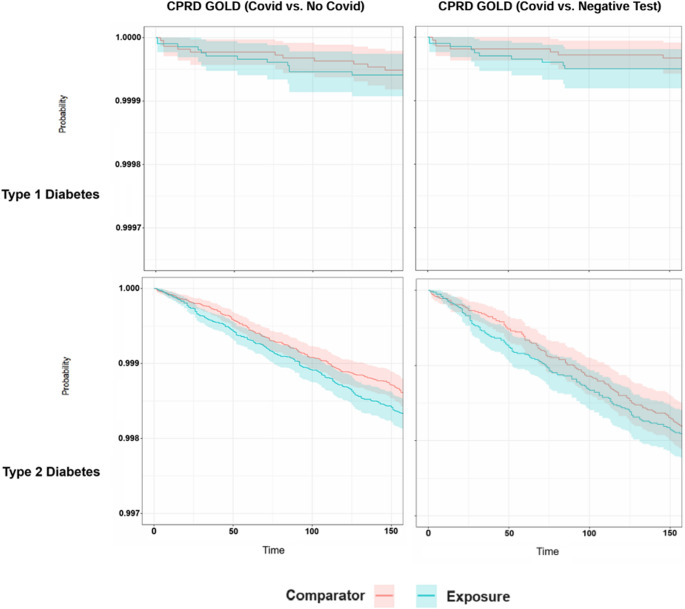
Kaplan-Meier curves (Overall) for Type 1 and Type 2 diabetes

**Fig. 6:**
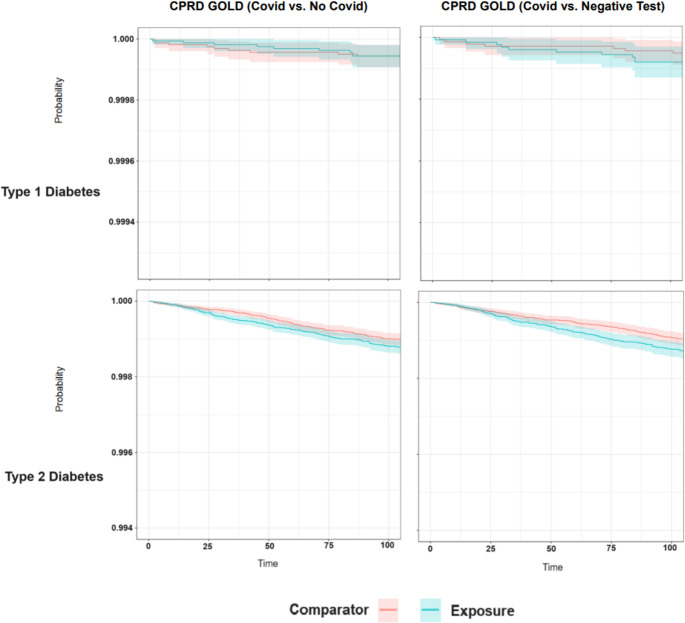
Kaplan-Meier curves (Female) for Type 1 and Type 2 diabetes

**Fig. 7:**
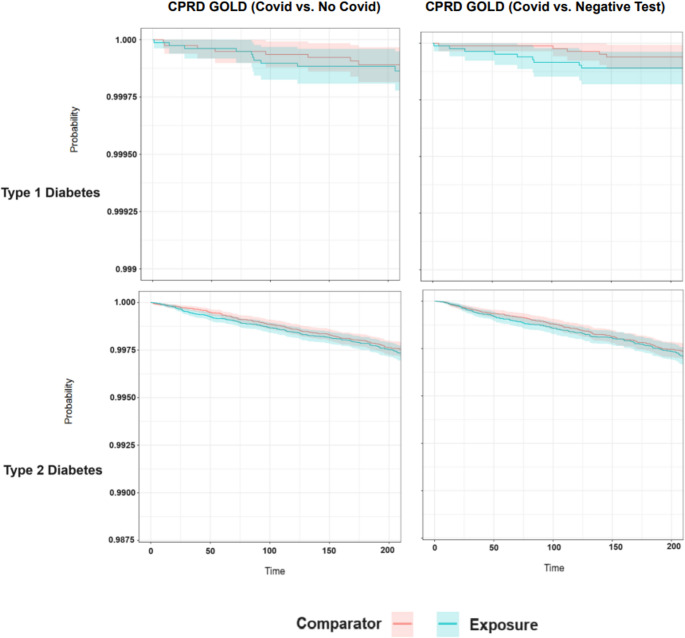
Kaplan-Meier curves (Male) for Type 1 and Type 2 diabetes

**Fig. 8:**
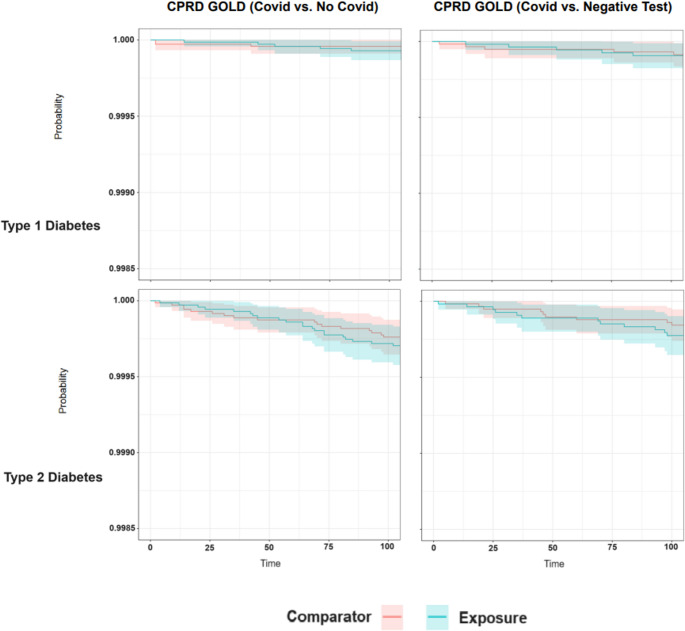
Kaplan-Meier curves (Age 19–39) for Type 1 and Type 2 diabetes

**Fig. 9:**
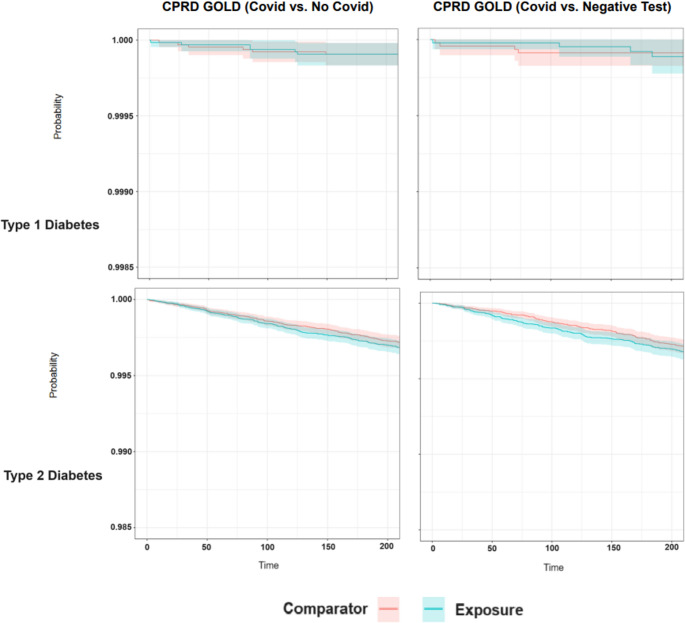
Kaplan-Meier curves (Age 40–59) for Type 1 and Type 2 diabetes

**Fig. 10:**
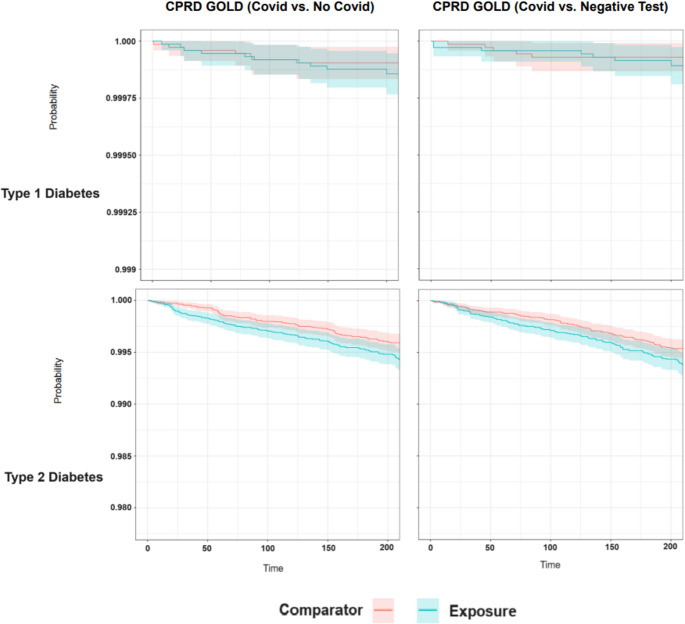
Kaplan-Meier curves (Age 60–79) for Type 1 and Type 2 diabetes

**Fig. 11:**
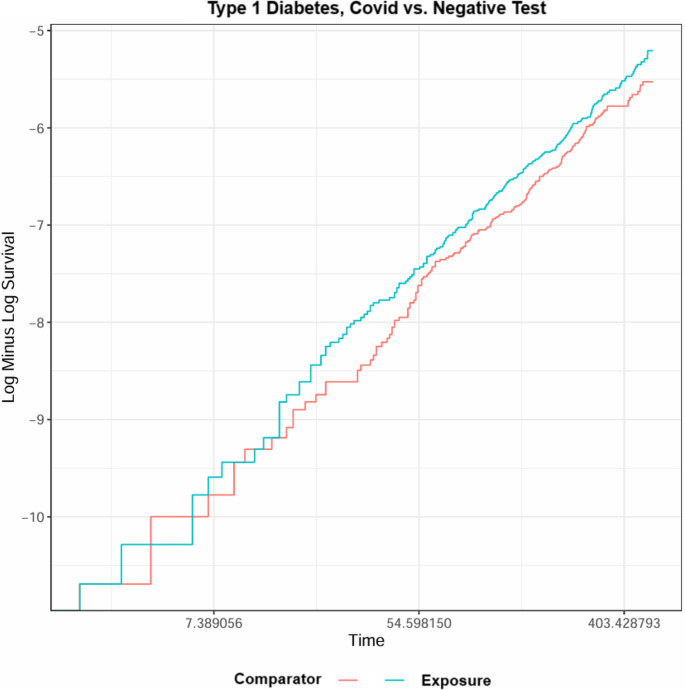
Log-log Kaplan-Meier curves for Type 1 diabetes, when matching Covid with No Covid

**Fig. 12:**
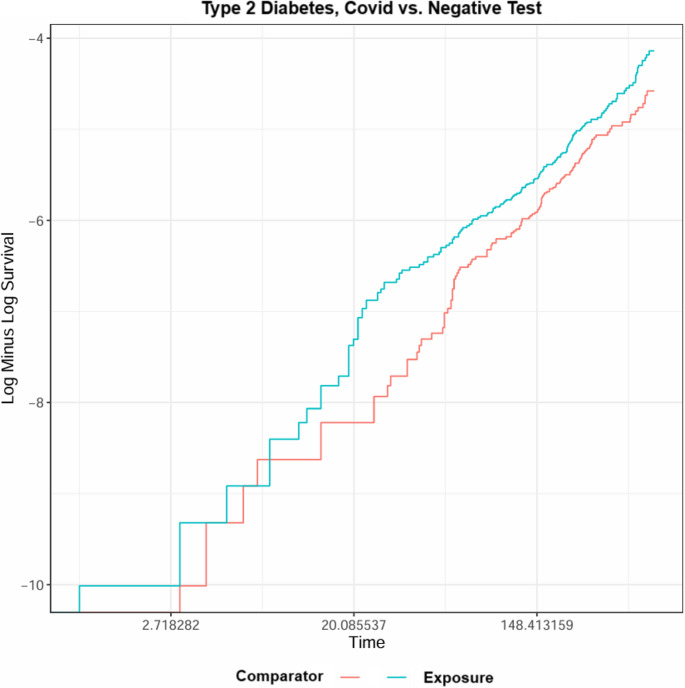
Log-log Kaplan-Meier curves for Type 2 diabetes, when matching Covid with No Covid

Kaplan-Meier plots for the development of Type 1 and Type 2 diabetes over time after index date stratified by exposure are available in the Appendix (Fig. 5). Note however that the log-log Kaplan-Meier curves were found to intersect early in the study period (Figs. 4, 5, 11 and 12 in the Appendix). We therefore include these results only for completeness and transparency. For Type 1 diabetes, we obtained an overall hazard ratio of 1.28 (0.64–2.37) when matching Covid and No Covid controls; and HR of 1.69 (0.68–2.93) when matching Covid with Negative tests (see Fig. 4 in the Appendix). For Type 2 diabetes, we obtained an overall hazard ratio of 1.08 (0.93–1.23) when matching Covid and No Covid; and HR of 1.04 (0.91–1.19) when matching Covid with Negative test (Fig. 4 in the Appendix). The Kaplan-Meier curves for matched cohorts stratified by age and sex can also be found in the Appendix (Figs. 6, 7, 8, 9 and 10).

### Discussion and Conclusions

Based on the results obtained for the matched cohorts in this study, we did not find a consistent association between SARS-CoV-2 infection and new onset of Type 1 diabetes. Similarly, we did not find an association between COVID-19 and the risk of developing Type 2 diabetes up to 1 year after infection. These findings were similar in sensitivity analyses using people who tested negative as controls, and across different age-sex strata and time periods.

Although there have been studies showing a 40% [8] or even a 2-fold increase [9] in the risk of developing Type 1 diabetes following SARS-CoV-2 infection, these studies were conducted without propensity score matching [8] or only in paediatric population [9], which may explain the differences in the results obtained.

One study utilising national databases of the US Department of Veterans Affairs demonstrated a 1.4-fold increased risk of incident diabetes in the post-acute phase of COVID-19 up to September 2021, with the majority of diagnoses being T2D [20]. Two meta-analyses together including 15 patient cohorts suggest increased risk between 1.59 and 1.64 for incident diabetes in COVID-19 patients in the post-acute phase, with the majority being T2D rather than T1D [21, 22].

The different findings obtained in our study could be related to the fact that we used population-based data from the UK NHS, and from a period with almost universal healthcare coverage and free testing in the community. The analysis of such data would be less likely to incur selection bias, as we would identify a large majority of diagnosed cases of COVID-19 in the study period of interest (a small proportion of cases could of course still be missed or undiagnosed for individuals in the population who were not routinely screened during this time period). Additionally, previous studies were likely affected by protopathic bias [23], where people were diagnosed with pre-existing diabetes immediately following infection as part of routine work upon admission. We conducted sensitivity analyses using test-negative controls and stratified by time to minimise this potential issue.

We only found one potentially relevant association, with Covid patients showing a 35% increased risk of Type 2 diabetes in the 31–90 days after infection when compared to matched Negative test participants. However, these results were not corroborated in the analyses comparing Covid with matched Covid-free controls, suggesting this could be a spurious finding.

### Limitations and Strengths

A limitation of this study (and of any study using real-world data) is that, until a disease has been diagnosed and entered into the database, we have to treat the absence of a diagnosis as absence of the disease. Thus, the No Covid control group for example may have included some patients with undiagnosed COVID-19 that had not yet been tested; however, this was likely a very small proportion of the participants, as SARS-CoV-2 testing was freely available in the UK during the study period. Thus, this limitation is unlikely to have had a significant effect on the results of our analysis.

A similar limitation is the possible time-lag between appearance of Type 1 or Type 2 diabetes in a patient, and the date of diagnosis by the GP. This could have some effect on the Poisson regression time-stratified analysis, as well as on the “survival times” in the Kaplan-Meier survival curves. However, this time-lag would likely affect Covid and No Covid patients equally, and thus would not have a significant impact on the results obtained in this study.

Strengths of the study include the large sample size, the universal coverage and representativeness of UK NHS health care records, and the ample free community testing available nationally during the study period. All these should reduce the selection bias likely present in previous studies.

Additionally, we used state-of-the-art large scale propensity score methods to minimise confounding, and ran sensitivity analyses to test the robustness of our findings. Finally, we followed open science principles, and have made all our analytical code and study findings available in the public domain.

## Conclusions

Based on the results obtained in this study, and in contrast to many previous publications, we did not find a significant association between SARS-CoV-2 infection and new onset of Type 1 or Type 2 diabetes. These findings were similar in our sensitivity analyses, and across different age-sex strata and time periods.

However, further studies need to be undertaken to understand the potential causal effect of SARS-CoV-2 infection on the risk of developing diabetes mellitus. Of particular interest is the possible association with development of Type 1 diabetes in paediatric populations, which was not investigated in the present study. 

## Supplementary Information

Below is the link to the electronic supplementary material.

Supplementary Material 1

## Figures and Tables

**Fig. 13:**
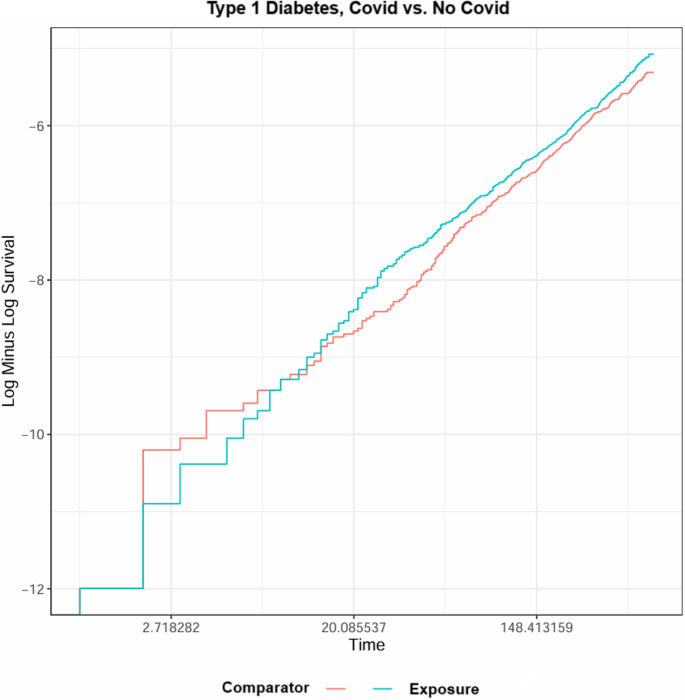
Log-log Kaplan-Meier curves for Type 1 diabetes, when matching Covid with No Covid

**Fig. 14:**
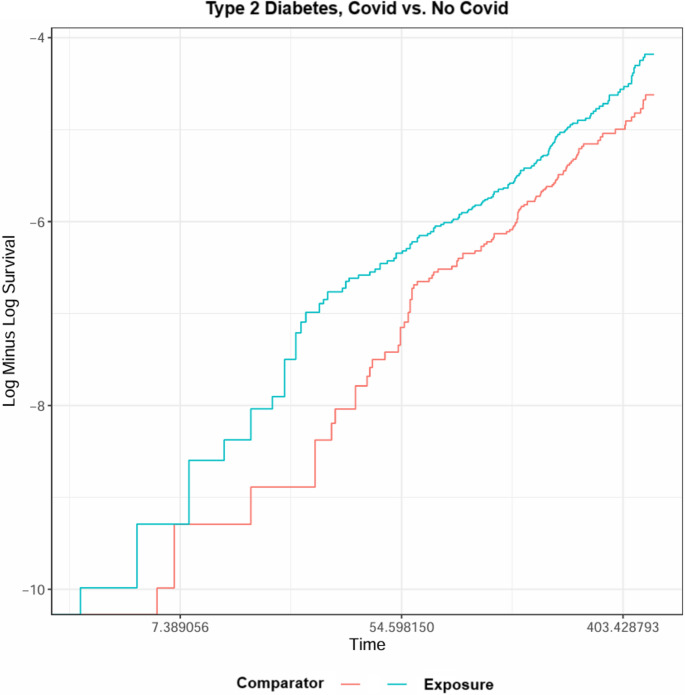
Log-log Kaplan-Meier curves for Type 2 diabetes, when matching Covid with No Covid

## Data Availability

The data underlying this study was the UK Clinical Practice Research Datalink (CPRD) GOLD database, release 2022/07 [15]. CPRD data were obtained under the CPRD multi-study license held by the University of Oxford after Research Data Governance approval.

## Appendix

**Table 3:** Characterisation of cohorts prior to matching

	Covid vs no covid		Covid vs Negative test	
	Covid	No Covid	Covid	Negative Test
Number of Subjects	193,692	2,080,978	193,692	367,201
Number of Records	193,692	2,080,978	193,692	367,201
Sex: Female	106,519 (55%)	1,037,173 (50%)	106,519 (55%)	206,526 (56%)
Male	87,173 (45%)	1,043,805 (50%)	87,173 (45%)	160,675 (44%)
Age: Median (q25-q75)	41 (30–54)	50 (35–65)	41 (30–54)	45 (33–58)
0 to 19	4,087 (2%)	25,764 (1%)	4,087 (2%)	5,763 (2%)
20 to 39	83,458 (43%)	644,049 (31%)	83,458 (43%)	135,204 (37%)
40 to 59	75,984 (39%)	714,746 (34%)	75,984 (39%)	144,174 (39%)
60 to 79	25,914 (13%)	551,767 (27%)	25,914 (13%)	71,511 (19%)
80 to 150	4,249 (2%)	144,652 (7%)	4,249 (2%)	10,549 (3%)
Comorbidities**:** Diabetes	0 (0%)	0 (0%)	0 (0%)	0 (0%)
HIV	96 (0%)	863 (0%)	96 (0%)	182 (0%)
Stroke	1,220 (1%)	21,224 (1%)	1,220 (1%)	2,840 (1%)
Myocardial infarction	1,631 (1%)	25,221(1%)	1,631 (1%)	3,614 (1%)
Inflammatory bowel disease	1,436 (1%)	12,147 (1%)	1,436 (1%)	2,876 (1%)
Osteoporosis	1,821 (1%)	39,805 (2%)	1,821 (1%)	5,021 (1%)
Rheumatoid arthritis	1,054 (1%)	12,280 (1%)	1,054 (1%)	2,238 (1%)
Chronic liver disease	334 (0%)	5,141 (0%)	334 (0%)	781 (0%)
Chronic kidney	3,662 (2%)	67,312 (3%)	3,662 (2%)	8,292 (2%)
Heart failure	1,026 (1%)	17,615 (1%)	1,026 (1%)	2,355 (1%)
Venous thromboembolism	3,312 (2%)	39,771 (2%)	3,312 (2%)	6,855 (2%)
Hypothyroidism	5,531 (3%)	64,128 (3%)	5,531 (3%)	11,577 (3%)
Depressive disorder	29,521 (15%)	244,982 (12%)	29,521 (15%)	54,525 (15%)
Anxiety	37,052 (19%)	297,791 (14%)	37,052 (19%)	66,061 (18%)

**Table 4:** Patient counts for COVID-19 cases, Type 1 and Type 2 diabetes in the database prior to matching

	No Covid or Negative Test			Covid		
	*N*	Type 1 Diabetes	Type 2 Diabetes	*N*	Type 1 Diabetes	Type 2 Diabetes
(Covid vs. No Covid)	2,080,978	252	5,714	193,692	26	522
(Covid vs. Negative Test)	367,201	54	1,352	193,692	26	522

## Declarations

### Reporting of Results

This work adheres to STROBE guidelines for reporting observational studies [24]. A completed STROBE checklist can be found in the Supplementary Materials.
